# The impact of sarcopenia on the progression of chronic non-bacterial osteomyelitis

**DOI:** 10.1186/s13244-025-02001-w

**Published:** 2025-06-04

**Authors:** Daniel Vogele, Janine Akbulut, Franziska Müller-Reichart, Aleš Janda, Henner Morbach, Hermann J. Girschick, Matthias C. Schaal, Meinrad Beer, Clemens Benoit

**Affiliations:** 1 Department of Diagnostic and Interventional Radiology, University Hospital Ulm, Ulm, Germany; 2 Department of Diagnostic and Interventional Radiology, University Hospital Würzburg, Pediatric Radiology, Würzburg, Germany; 3 Department of Pediatrics and Adolescent Medicine, University Hospital Ulm, Ulm, Germany; 4 Department of Pediatrics, University Hospital Würzburg, Würzburg, Germany; 5 Children´s Hospital, Vivantes Klinikum im Friedrichshain, Berlin, Germany

**Keywords:** Chronic non-bacterial osteomyelitis, Sarcopenia, MRI, CNO-lesions, Autoinflammation

## Abstract

**Objectives:**

Chronic non-bacterial osteomyelitis (CNO) is the most common autoinflammatory bone disease in children and adolescents. This study investigated the progression of CNO lesions during therapy and the potential impact of sarcopenia on disease progression, utilizing routine MRI.

**Methods:**

A retrospective analysis of MRI examinations was conducted on 29 children and adolescents with CNO. CNO lesions were segmented. Sarcopenia was assessed using the total psoas muscle index (PMI) at lumbar vertebral levels L3/4 and L4/5. Measurements were taken at four time points during the disease course (T1: baseline, T2–T4: follow-up). Based on the PMI, patients were classified as sarcopenic or non-sarcopenic, and the progression of CNO lesions and the impact of sarcopenia were analyzed.

**Results:**

A total of 29 patients, aged 1–16 years, were included in the study, with 13 males and 16 females. Patients with sarcopenia had a significantly larger mean lesion area (868.95 mm^2^, SD = 684.49) compared to those without sarcopenia (636.11 mm^2^, SD = 832.41); *p* = 0.042, *d* = 0.4). The comparison between the two patient groups revealed a consistently lower percentage reduction in lesion size for the sarcopenic patients at all time points. Notably, the difference between T1 and T3 was statistically significant (*p* = 0.045, *d* = 0.82).

**Conclusion:**

The present study indicates that sarcopenia may serve as a negative prognostic factor in the treatment of CNO. Incorporating sarcopenia assessment as an additional parameter in routine whole-body MRI examinations could enhance the evaluation process.

**Critical relevance statement:**

Sarcopenia can be assessed using routine whole-body MRI in patients with CNO and may serve as a negative prognostic factor, potentially enhancing the evaluation process.

**Key Points:**

- Whole-body MRI is crucial for diagnosing and monitoring CNO.
- Routine whole-body MRI in CNO patients can also be used to assess sarcopenia as an additional parameter.
- Sarcopenia may act as a negative prognostic factor in CNO treatment, potentially improving the evaluation process.

**Graphical Abstract:**

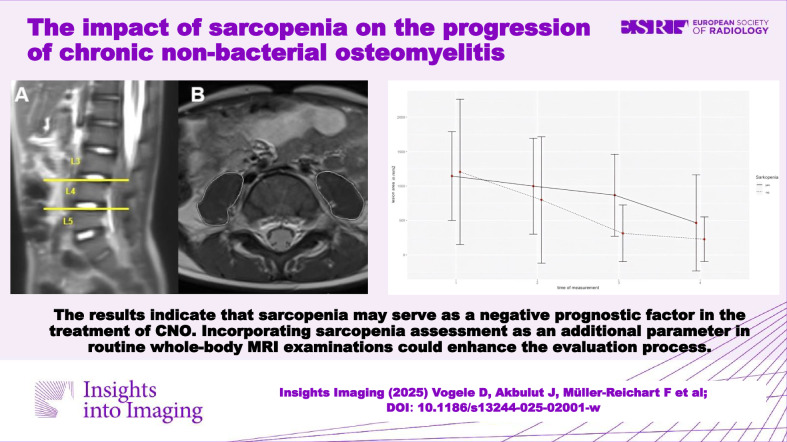

## Introduction

Chronic non-bacterial osteomyelitis (CNO) and its most severe form, chronic recurrent multifocal osteomyelitis (CRMO), are autoinflammatory bone disorders primarily affecting children and adolescents [1–4]. The prevalence is estimated to be 1 in 100,000 to 1,000,000, with an estimated annual incidence of approximately 4 per 1,000,000 according to a surveillance study. Despite its rarity, CNO is likely the most common autoinflammatory bone disease in childhood [1, 5–7]. The exact pathogenesis of CNO remains unknown. The hypothesis that infections might sustain the disorder was not supported by extensive microbiological analyses or the ineffectiveness of antibiotic treatment [3, 8]. Clinical presentation and severity of CNO vary significantly among patients, ranging from asymptomatic or mild inflammation of single bones to CRMO, sometimes leading to bone destruction. The most commonly affected areas are the metaphyses of long bones, pelvic bones, vertebral column, mandible, and shoulder girdle/clavicle [9, 10].

Cultures of blood and bone in CNO patients are invariably negative, and advanced assays have failed to identify any microbial cause. The prevalence of autoantibodies, such as antinuclear antibodies and rheumatoid factor, as well as the carriage of the human leukocyte antigen-B27 (HLA-B27) allele, is similar in both CNO patients and healthy individuals. Currently, no specific biomarkers exist for diagnosing CNO or predicting flare-ups [3, 10]. Proposed diagnostic criteria for CNO have yet to receive international validation and acceptance [11].

Imaging plays a crucial role in both diagnosing CNO and excluding differential diagnoses, as well as in monitoring treatment progress. CNO lesions may appear as radiolucent, osteolytic, or sclerotic on plain radiographs. However, in early stages, plain radiographs often show no characteristic changes [12, 13]. CNO is a systemic disorder that can affect multiple skeletal sites, making whole-body magnetic resonance imaging (MRI) highly sensitive and particularly useful [2, 14–16]. MRI can detect bone marrow edema in early disease stages, even before the development of bone erosions or sclerosis. Whole-body MRI, especially with fat-saturated T2-weighted sequences using inversion recovery (e.g., turbo inversion recovery magnitude (TIRM) or short tau inversion recovery (STIR) can identify inflammatory bone lesions and possible periosseous involvement [3, 17]. While gadolinium-enhanced T1 sequences are not routinely acquired, they can be helpful in clarifying and interpreting ambiguous findings. At diagnosis, whole-body MRI is important for identifying clinically silent lesions, particularly in the vertebral column. During follow-up, MRI is important for assessing disease activity and monitoring disease-associated sequelae, such as fractures or involvement of surrounding soft tissue [11].

Due to the unclear pathogenesis of CNO, targeted treatment is challenging. This challenge is compounded by the absence of randomized controlled trials, meaning treatment strategies rely solely on retrospective reviews and case series [10, 18]. The disease course is heterogeneous, with many prognostic factors under discussion, such as multifocal disease, inflammation of the pelvis or femur at disease onset, and high erythrocyte sedimentation rate [19] at baseline [20]. Recently, sarcopenia has gained attention as a prognostic factor in various diseases, not only in adult medicine but also in pediatric and adolescent medicine [21–24]. In the present study, the course of CNO lesions under therapy and the potential influence of sarcopenia were investigated using MRI.

Based on these findings, we hypothesized that:

1. In patients with CNO, sarcopenia can be detected using whole-body MRI, which is initially performed for diagnosis, follow-up, and monitoring.
2. Sarcopenia is a negative prognostic factor for the course of CNO lesions under therapy.

## Materials and methods

### Study population and imaging

In this study, MRI datasets from 48 patients diagnosed with CNO from two tertiary hospitals were retrospectively analyzed. The aim was to investigate the relationship between the severity of the main lesions and sarcopenia in children and adolescents with CNO. All patients were pseudo-anonymized and referred to as patient 1–48. Patients required whole-body MRI scans or at least abdominal/pelvic scans displaying the psoas muscle at the lumbar level to define sarcopenia. MRI examinations were performed using a 1.5-Tesla scanner (Magnetom Aera and Avanto, Siemens Healthcare) and a 3-Tesla scanner (Magnetom Skyra, Siemens Healthcare). Sequence parameters are detailed in Table 1. The image data was archived for analysis in the picture archiving and communication system (PACS) (DeepUnity Diagnost 2.0.2.2, Dedalus Healthcare Systems Group, Bonn). Patients older than 16 years, with incomplete examinations, poor image quality, or 3 or fewer MRI scans during the course of the disease were excluded. This resulted in a final study population of 29 patients.

**Table 1:** Sequence parameters for MRI acquisition were designed to ensure flexibility across patient cases

	T2 TIRM	T2 STIR	T1 TSE fs Dixon	T1 TSE
Matrix	320 × 224	320 × 224	384 × 307	448 × 186
Orientation	Coronal/axial	Coronal/axial	Coronal	Axial
Slice thickness	3 mm	6 mm	6 mm	4 mm
Time of repetition (TR)	3640 ms	6000 ms	597 ms	422 ms
Echo time (TE)	62 ms	81 ms	13 ms	11 ms
Flip angle	150	146	180	160
Time of inversion	220 ms	160 ms	–	–
Contrast media	–	–	–	Optional

While not all sequences were acquired for every patient, each examination included at least a T2-weighted TIRM or T2-weighted STIR sequence in the coronal orientation, accompanied by a T1-weighted sequence. Contrast-enhanced sequences were optionally included to facilitate a more detailed evaluation of specific regions as needed. If axial images for psoas muscle segmentation were not acquired, thin-slice coronal images were used to generate reconstructed axial views

*TIRM* turbo inversion recovery magnitude, *STIR* shot time inversion recovery, *TSE* turbo spin echo, *FS* fat saturation

#### CNO and sarcopenia

**Fig. 1:**
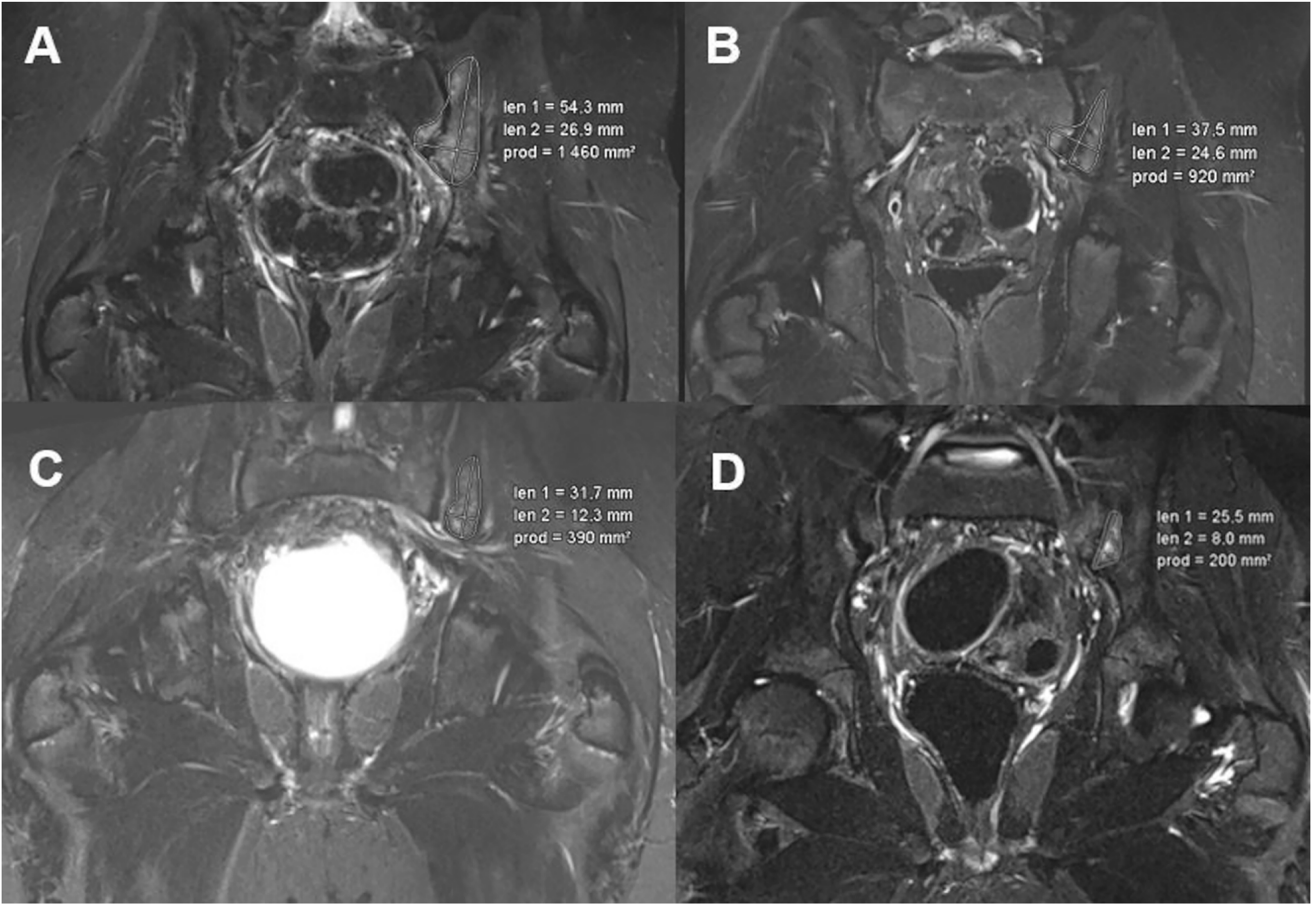
Example of a 9-year-old male patient with CNO. **A** displays a section of the coronal T2 TIRM sequence of the pelvis and proximal femora from the baseline examination (t1), revealing a CNO lesion extending across the sacroiliac joint. **B**–**D** depict subsequent examination time points (t2, t3, and t4), demonstrating a progressive reduction in the size of the CNO lesion over time

All MRI images of the patients were viewed and categorized into baseline (T1), follow-up 1 (T2) after 6 months, follow-up 2 (T3) after 12 months, and follow-up 3 (T4) after 18 months. For data collection on CNO, all radiologically detectable lesions, were noted, and one “target lesion” was defined. Lesions were mapped and measured in terms of surface area and maximum longest diameter at each time point. Figure 1 shows an example of a patient with CNO and a left pelvic lesion.

**Fig. 2:**
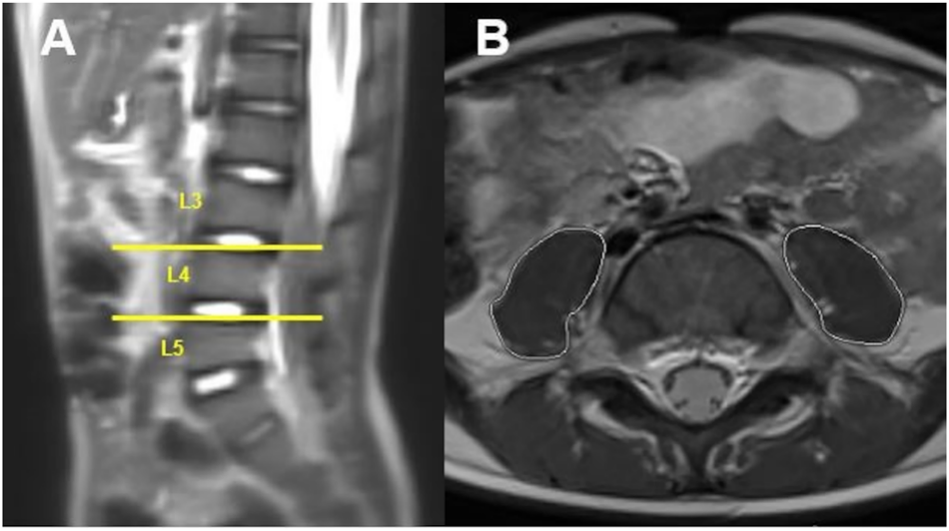
Example of a 5-year-old male patient with CNO. Lumbar levels were identified using sagittal images from the localizer (**A**), where the L3/4 and L4/5 intervertebral levels were marked (yellow lines). On the corresponding axial images (**B**) at these lumbar levels, the psoas muscle areas (PMA) on both sides were evaluated. **B** provides an example of the PMA determination at the L4/5 level (white lines)

For the classification of sarcopenia, MRIs were viewed in axial view at lumbar vertebral levels L3/4 and L4/5. The total psoas muscle area (tPMA) was measured at these levels using a PACS tool (freehand region of interest measurement tool). The sum of the right and left tPMA was calculated for both lumbar levels and expressed in mm^2^. Figure 2 shows an example of the determination of the tPMA. The *z*-scores were calculated by using a dedicated online tool based on recently published pediatric reference values for tPMA (https://ahrc-apps.shinyapps.io/sarcopenia/ [25]). The *z*-score calculator is only applicable for children and adolescents between 1 and 16 years, which is why 2 patients older than 16 years had to be excluded. Sarcopenia was defined as a *z*-score under −2. All variables were recorded at all available measurement points, but at least two.

### Statistics

The normal distribution of the data was assessed graphically using histograms and QQ plots and statistically using the Shaphiro–Wilk test. Variance homogeneity was evaluated using the Levene test and Box’s M test. The variables were described using mean, median, and quantiles/quartiles. Differences between the sarcopenic and non-sarcopenic patients were analyzed using mixed-model ANOVA. Due to the lack of sphericity indicated by Mauchly´s test, the effect size was reported using Greenhouse-Geisser-corrected values. Individual *p*-values were calculated using independent t-tests, followed by a post-hoc power analysis.

The correlation between lesion area and maximum diameter was assessed using Spearman’s rank correlation coefficient. Missing data were excluded from the analysis. Data analysis was conducted using R and R Studio software (version 2023.12.1 + 402, Posit Software, PBC), and the power analysis was performed with G*Power (version 3.1.9.7, Heinrich Heine Universität Düsseldorf).

Ethical approval for this study was obtained from the local ethics committee of both sites (no. 349/21 and 68/03).

#### Clinical parameters

The clinical data on the progression of the disease and treatment for all patients were documented using each clinic’s own system. This documentation covered various therapies, including drug treatments and their durations. The drug treatments included analgesics, non-steroidal anti-inflammatory drugs (NSAIDs), antibiotics, and immunosuppressants.

## Results

After applying the specified exclusion criteria, 29 patients remained in the study. Among these, 13 were male (44.83%) and 16 were female (55.17%). The children and adolescents had an average age of 11 years (range 1–16 years; SD = 3.88). Within this group, 10 patients, 4 males (13.78%) and 6 females (20.69%) were sarcopenic, and 19, 9 males (31.03%) and 10 females (34.48%) patients were non-sarcopenic. Patient characteristics are detailed in Table 2.

**Table 2:** Patient characteristics and groups of sarcopenic/non-sarcopenic patients

Characteristics	Patients with sarcopenia (*n* = 10)	Patients without sarcopenia (*n* = 19)
Female	*n* = 6, 60%	*n* = 10, 52.63%
Male	*n* = 4, 40%	*n* = 9, 47.37%
Age total	11.1 ± 4.82	10.63 ± 3.42
Age female	12.33 ± 4.32	9.6 ± 3.13
Age male	9.25 ± 5.56	11.78 ± 3.53

**Fig. 3:**
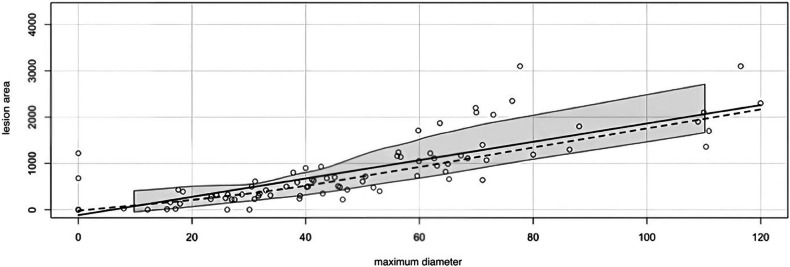
Diagram of the correlation between the maximum diameter and the lesion area. Both in mm^2^

The correlation between the area of the lesion and the maximum diameter was tested using Spearman´s test, yielding a statistically significant result (*p* < 0.001, *r* = 0.87). Given this strong correlation, only the lesion area was used in subsequent calculations (Fig. 3).

On average, the size of the CNO lesions continuously decreased under treatment during the observation period. The average area (in mm²) of the CNO lesions was 1184.72 (SD = 922.22) at T1, 867.14 (SD = 841.78) at T2, 504.14 (SD = 542.54) at T3 and 308.31 (SD = 486.64) at T4. The reduction in lesion size was statistically significant for all time points with a *p*-value of less than 0.001 and an effect size (η²) of 0.267.

Overall, the mean lesion area for patients with sarcopenia was 868.95 (SD = 684.49), which was greater than the 636.11 (SD = 832.41) for patients without sarcopenia. This difference between the two groups was statistically significant (*p* = 0.042, *d* = 0.4).

Within the group of sarcopenic patients, there was a continuous reduction in lesion size from T1 to T4. The size decreased from 1145.00 (SD = 646.57) at T1 to 998.80 (SD = 697.09) at T2, a reduction of 12.77%. By T3, the size further decreased to 867.00 (SD = 595.35), a 24.28% reduction from T1. By T4, the lesion size had reduced by 59.36%, reaching 465.00 (SD = 697.94).

The non-sarcopenic patients also showed significant reductions in lesion size. Their average lesion size decreased from 1205.63 mm² at T1 to 798.26 mm² at T2, a reduction of 33.79%. By T3, the size was further reduced to 313.16 mm², a 74.05% reduction from T1. Finally, by T4, the lesion size decreased to 227.37 mm², representing a reduction of 81.14%.

The comparison between the two patient groups showed a consistently lower percentage reduction in lesion size for the sarcopenic patients at all time points (T1–T2: 12.77% vs 33.79%; T1–T2: 24.28% vs 74.05%; Time point 1–4 (T1–T4): 59.36% vs 81.14%). The difference in lesion reduction between T1 and T3 was statistically significant (*p* = 0.045, *d* = 0.82). No significant differences were found in lesion reduction at the other time points.

**Fig. 4:**
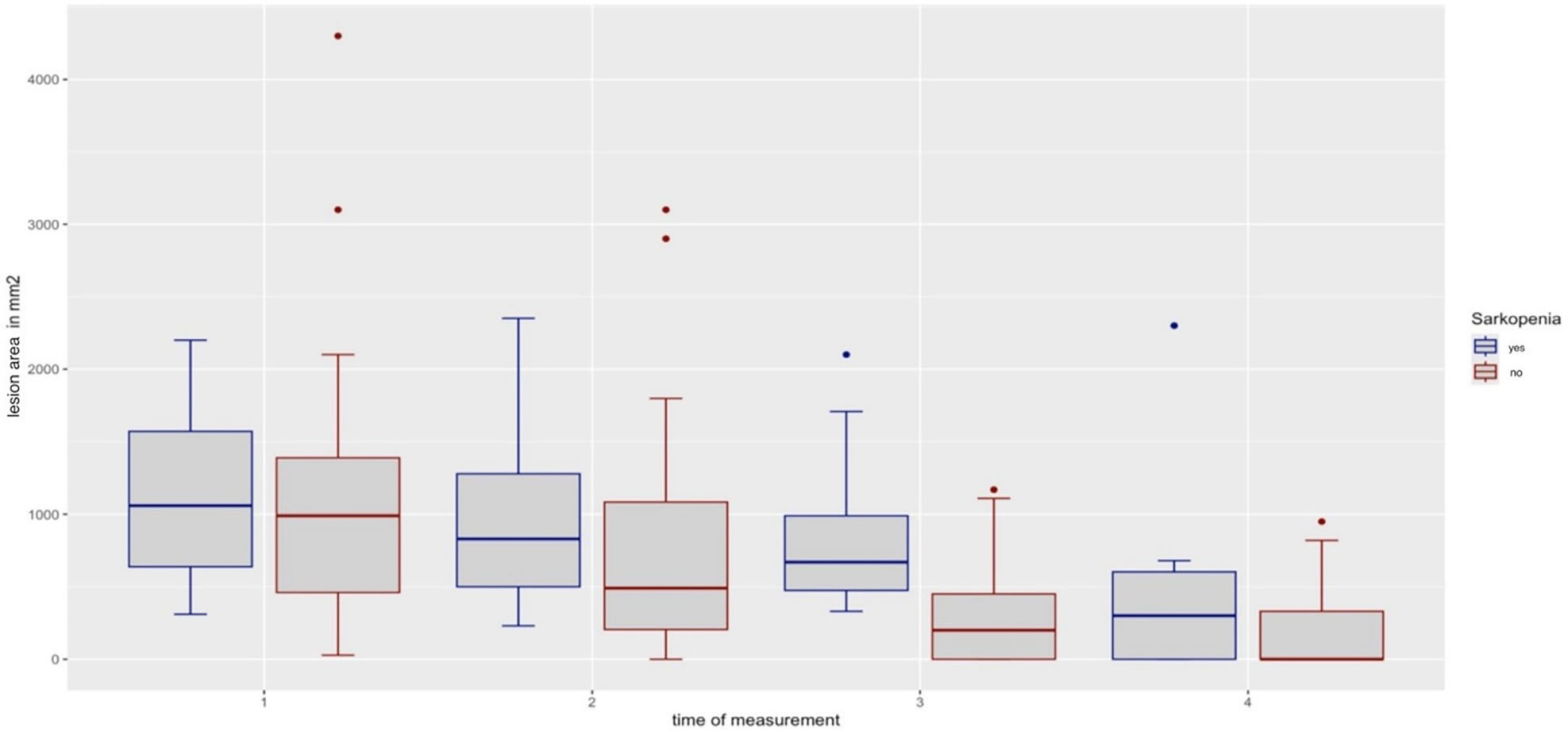
Boxplot representation of the lesion at the four measurement times (T1–T4) for sarcopenic (blue) and non-sarcopenic patients (red)

**Fig. 5:**
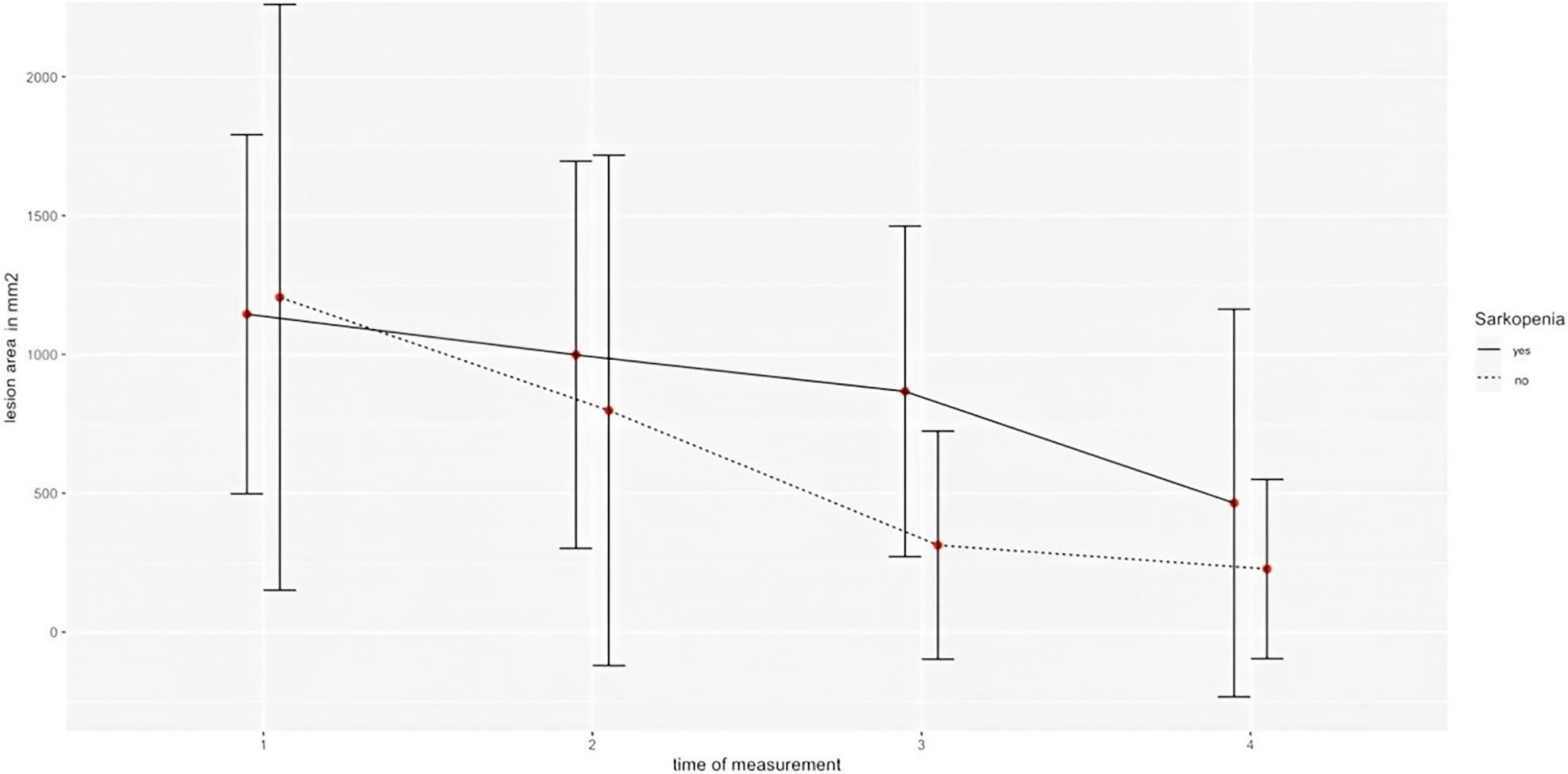
Progression of the lesion area over the four measurement times (T1–T4) in a line diagram, differentiated according to sarcopenic (straight) and non-sarcopenic (dotted) patients

The change in lesion sizes from T1 to T2, T2 to T3, and T3 to T4, separately for sarcopenic and non-sarcopenic patients, is illustrated in Figs. 4 and  5.

No significant difference was found between lumbar vertebral levels L3/4 and L4/5 when determining the tPMA, as the score takes both into account. Due to the very small differences in the determination of tPMA at the two anatomical localizations, there were no different classifications of the patients, as sarcopenic or score was determined. A post hoc calculation of the statistical power yielded a value of 0.89, indicating a high reliability of the results.

## Results of clinical data

All patients received NSAIDs with additional, varying therapy in many cases, e.g., non-biologic disease-modifying anti-rheumatic drugs, tumor necrosis factor α inhibitors, or bisphosphonates. This applies to both the patients in both clinics. Corticosteroids are occasionally used for acute symptom control, though long-term use is limited due to side effects. Emerging therapies targeting specific inflammatory pathways, such as IL-1 or IL-6 inhibitors, are also being explored to improve outcomes in resistant cases. The differences in the treatment approaches are due to different initial diagnosis times and the current absence of specific guidelines.

## Discussion

To the best of our knowledge, no previous analysis has examined sarcopenia in children and adolescents with CNO. Our study revealed that patients with sarcopenia had significantly larger inflammatory lesions and experienced a smaller reduction in lesion size during therapy. The role of sarcopenia as a consequence of malnutrition in inflammatory states and change of inflammatory biomarkers is well established, not only in elderly patients [26–29]. Our findings suggest that sarcopenia can serve as a negative prognostic indicator in CNO, particularly regarding lesion development and potential outcomes in pediatric patients. We propose that tPMA analysis be integrated into the evaluation process using MRI scans already performed for routine diagnostics and follow-up, thereby avoiding the need for additional imaging or patient burden.

Although the European Working Group on Sarcopenia in Older People (EWGSOP) has recently provided a clear definition of sarcopenia in adults, there is currently no standardized method for diagnosing sarcopenia in children [30–32]. According to the EWGSOP, muscle strength may be a more reliable marker of adverse outcomes in elderly individuals, however, comprehensive data for children are lacking [33, 34]. Measuring muscle strength is particularly challenging in children under 5–6 years of age due to limited compliance and the absence of established reference values for traditional tests, such as grip strength and the 6-min walking distance [35, 36].

To assess skeletal muscle loss, various measures derived from cross-sectional imaging (such as computed tomography (CT) or MRI) are used, including tPMA, psoas muscle index (PMI), skeletal muscle index (SMI), and dorsal muscle area [37–42]. In children, tPMA seems to be a robust, objective, reliable, and easily measurable marker of clinical nutrition [31, 37, 39, 43]. This is further supported by recent research challenging the suitability of the psoas muscle as a sentinel muscle for assessing sarcopenia [44, 45]. tPMA, derived from single-slice cross-sectional images at lumbar levels L3–4 and L4–5, is widely recognized as a direct marker of sarcopenia in children and has also been validated in adults [35]. It has been utilized for skeletal muscle assessment in numerous studies, including those involving children with oncologic diagnoses, Crohn’s disease, pediatric intestinal transplants, and infants undergoing liver transplantation [23, 24, 46–49]. Moreover, tPMA measurements obtained via CT and MRI are comparable, with an intraclass correlation coefficient of 0.98, highlighting their consistency across imaging modalities [50].

Other validated techniques for assessing sarcopenia include dual-energy X-ray absorptiometry (DEXA), bioelectric impedance analysis (BIA), and quantitative CT measurements [21]. However, both DEXA or BIA require additional examinations, potentially increasing the burden on patients. Furthermore, pediatric reference values for these methods are only available for children aged 3 years and older (DEXA) or 5 years and older (BIA) [51, 52]. Concerns about additional radiation exposure (e.g., DEXA or even CT scans), potential need for sedation (MRI scans), or the high costs associated with CT and MRI are significant considerations when evaluating sarcopenia assessment methods, particularly for young oncological patients. In children with oncological diagnoses, sectional images are often already available and can be repurposed for opportunistic sarcopenia assessment, eliminating the need for additional imaging. Similarly, in children and adolescents with CNO, whole-body MRI is a standard tool for diagnosis and monitoring disease progression. These existing scans can also be utilized to assess sarcopenia without imposing any further burden on the patient.

Lurz et al published age- and gender-specific reference values for tPMA at the L3–4 and L4–5 lumbar levels, accompanied by a freely available online *z*-score calculator tool [25]. As mentioned earlier, additional assessment of sarcopenia imposes no extra burden or active effort on the patient, making it a valuable source of opportunistic diagnostic information. tPMI can be calculated at different lumbar levels, as the original method by Lurz et al incorporates measurements from L3–4 and L4–5 [25]. In our study, we observed only minimal variations between these two levels, which did not affect the diagnosis of sarcopenia. This aligns with findings from other studies, which demonstrated that sarcopenia assessments at different anatomical sites reliably reflect the total psoas muscle volume.

In pediatric patients, cross-sectional imaging—particularly MRI—is commonly used for skeletal muscle analysis, as functional criteria such as muscle strength are challenging to assess in younger children due to limited compliance. Standard adult treatments for sarcopenia, including exercise programs, nutritional interventions, or pharmacological therapies (e.g., anabolic steroids or ghrelin agonists), are often unsuitable for children. Instead, tailored approaches that consider their developmental stage and specific needs are required.

To address these challenges, standardized diagnostic protocols for pediatric sarcopenia must be developed, alongside expanded research beyond oncological contexts, such as CNO. This should also include additional MR-sequences for optimal assessment of sarcopenia, such as axial T1- or T2-weighted images at least for the abdomen. With such a protocol also other parameters for body composition analysis, such as total muscle area, visceral adipose tissue, or subcutaneous adipose tissue could be evaluated. These efforts could deepen our understanding of the impact of sarcopenia in children and facilitate the creation of targeted therapies and interventions specifically designed for pediatric and adolescent populations.

The findings of this study aim to address the aforementioned aspects of sarcopenia in patients with CNO, providing a foundation for future research.

### Limitations

Our retrospective study has several limitations. Firstly, the dual-center design limits the generalizability of the findings. Additionally, the relatively small study sample size, largely due to the rarity of the disease [1, 2], limits the statistical power. Another limiting factor is the variability of therapeutic approaches. Given the absence of standardized treatment guidelines for CNO, participants received different therapies [53]. This complicates the comparison of the results. Individual variations in treatment may have influenced the disease, further challenging the interpretation of the data.

### Prospects

As personalized medicine becomes increasingly important, it is essential to consider negative prognostic factors [54]. Sarcopenia is not only a clinical condition characterized by the loss of muscle strength and mass, but it also significantly affects the patients´ quality of life [33]. This can have a profound impact on patients. By diagnosing sarcopenia and combining it with clinical and laboratory parameters, a prognostic score could be developed. This could enable more targeted treatment strategies for children and adolescents with CNO, potentially altering the course of their disease.

### Conclusion

Our findings demonstrate a continuous reduction in lesions under therapy among children and adolescents with CNO. Since the CNO lesions were larger in patients with sarcopenia, the sarcopenia may also be due to a longer or more severe course of the disease. However, patients with sarcopenia exhibit a slower rate of lesion reduction and an overall smaller decrease in lesion size (Figs. 3 and 4). This indicates that sarcopenia is a significant negative prognostic factor influencing the course of CNO.

Similar observations have been reported in other studies for various diseases where sarcopenia was identified as a negative factor. For instance, it has been shown to negatively affect outcomes in children and adolescents with hepatoblastoma and neuroblastoma [22, 23]. These observations align with our conclusion that sarcopenia may play a substantial role in the progression of CNO. To validate these results and further understand the impact of sarcopenia, multicenter prospective studies with larger cohorts are essential.

## Data Availability

The datasets generated or analyzed during the study are available from the corresponding author upon reasonable request.

## Abbreviations

BIA: Bioelectric impedance analysis
CNO: Chronic non-bacterial osteomyelitis
CRMO: Chronic recurrent multifocal osteomyelitis
CT: Computed tomography
DEXA: Dual-energy X-ray absorptiometry
EWGSOP: European working group on sarcopenia in older people
MRI: Magnetic resonance imaging
NSAID: Non-steroidal anti-inflammatory drug
PACS: Picture archiving and communication system
PMI: Psoas muscle index
SD: Standard deviation
STIR: Short tau inversion recovery
T1–T4: Time point 1–4
TIRM: Turbo inversion recovery magnitude
tPMA: Total psoas muscle area
